# A Distinct Host-Microbiome Signature Underlies the Accelerated Malignant Potential of Colorectal Laterally Spreading Tumors

**DOI:** 10.1016/j.gastha.2026.101102

**Published:** 2026-08-21

**Authors:** Tymofiy Lutsiv, Henry J. Thompson, Oliver Fiehn, Melanie G. Gareau, Alexander D. Borowsky, Brian H. Chen, Hisham Hussan

**Affiliations:** 1 Cancer Prevention Laboratory, Colorado State University, Fort Collins, Colorado; 2 Graduate Program in Cell and Molecular Biology, Colorado State University, Fort Collins, Colorado; 3 West Coast Metabolomics Center, University of California, Davis, California; 4 Department of Anatomy, Physiology, and Cell Biology, University of California, Davis, California; 5 Department of Pathology and Laboratory Medicine, University of California, San Francisco, San Francisco, California; 6 Helen Diller Family Comprehensive Cancer Center, University of California, San Francisco, San Francisco, California; 7 Department of Pathology and Lab Medicine, University of California, Davis, Sacramento, California; 8 Center for the Science of Aging, Sutter Health Research Institute, San Francisco, California; 9 Division of Gastroenterology, Department of Internal Medicine, University of California, Davis, Sacramento, California; 10 Davis Comprehensive Cancer Center, The University of California, Sacramento, California

**Keywords:** Laterally Spreading Tumors, Transcriptome, Microbiome, Fusobacterium, Cellular Senescence

## Abstract

**Background and Aims:**

Laterally spreading tumors (LST) are flat colorectal neoplasms with an accelerated risk of malignant transformation and interval colorectal cancer. Despite their clinical importance, the molecular and microbial mechanisms underlying LST's aggressive biology remain poorly understood. Thus, we aimed to characterize the transcriptomic and microbial landscape of LST in comparison with paired protruding lesions and the adjacent normal colonic tissue.

**Methods:**

Formalin-fixed, paraffin-embedded tissues from 36 samples were obtained from 15 adults and analyzed using RNA sequencing and 16S rRNA gene amplicon sequencing. Patterns of the differential gene expression were assessed using Gene Set Enrichment Analysis and Ingenuity Pathway Analysis. Microbial community composition and its predicted functional capacity were evaluated with analysis of compositions of microbiomes with bias correction in QIIME 2 and Phylogenetic Investigation of Communities by Reconstruction of Unobserved States 2, respectively.

**Results:**

Compared with paired protruding lesions and normal tissue, LST exhibited a distinct protumorigenic transcriptomic profile marked by the activation of MYC, E2F, mTOR, DNA damage, and senescence-associated secretory phenotype pathways, as well as robust proinflammatory signaling driven by TNF, NF-κB, IL-1, and IL-17. LST tissue also demonstrated a permissive environment for genomic instability. Microbiome analysis revealed enrichment of *Fusobacterium* and depletion of beneficial taxa, including *Lactococcus*, accompanied by predicted suppression of carbohydrate fermentation and short-chain fatty acid production, as well as altered sulfur metabolism. *Fusobacterium* abundance correlated with increased TNF expression, supporting a microbiota-driven inflammatory niche in LST.

**Conclusion:**

LST are characterized by a unique inflammatory/metabolic/senescence axis that distinguishes them from other paired colorectal tissue samples. This procarcinogenic signature is driven by a *Fusobacterium*-enriched and carbohydrate-fermentation-depleted microbial ecosystem. These findings highlight the gut microbial ecosystem as a critical cofactor in LST pathogenesis and further support that combined host/microbiota-targeted strategies may improve colorectal cancer prevention in this population. Given the exploratory nature and limited cohort size, these findings require validation in larger prospective cohorts with metagenomic and metabolomic integration.

## Introduction

Colorectal cancer (CRC) represents the most prevalent gastrointestinal cancer worldwide, imposing a substantial clinical and economic burden. About 5% to 7% of adults harbor high-risk flat polyps, defined as laterally spreading tumors (LST).^1^^,^^2^ LST were first described by Kudo^3^ as nonprotruding lesions measuring >20 mm in diameter characterized by lateral expansion along the luminal wall with a low vertical axis. Compared to protruding lesions (PL), LST carry a higher risk of malignancy, local recurrence, synchronous or metachronous neoplasia, and faster progression to CRC.^4^, ^5^, ^6^ The LST flat morphology also makes them difficult to detect during colonoscopy, contributing to interval CRC, while LST endoscopic resection is technically challenging and associated with increased risks of recurrence and perforation.^2^^,^^7^^,^^8^ Finally, LST are more common in adults older than 65 years—an age group at higher risk of colonoscopy complications and less likely to be screened for CRC after the age of 75.^9^, ^10^, ^11^, ^12^, ^13^ Yet, the molecular mechanisms underlying the aggressive biology of LST remain poorly understood. Bridging this knowledge gap could facilitate biomarker-driven LST surveillance and precision prevention strategies.

Most LST are composed of adenomas, yet their molecular features are unique compared to those of PL, suggesting a distinct flat-type genetic pathway. For instance, LST exhibit significantly higher mutation rates than PL—rates that are comparable to microsatellite-stable CRC, indicating a state of heightened genomic instability even in the preinvasive stage.^14^, ^15^, ^16^, ^17^ This mutation phenotype in LST is also seen with cellular senescence and increased replication errors, implying that these lesions are biological reservoirs of premature aging.^17^, ^18^, ^19^, ^20^, ^21^ Notably, cellular senescence induces a senescence-associated secretory phenotype (SASP)—a chronic localized and systemic proinflammatory state, hallmarked by dysregulated immune cells.^22^, ^23^, ^24^ Cellular senescence and SASP exacerbate CRC risk, likely by promoting stem cell division, replication errors, and somatic mutations via a persistent tumor-promoting microenvironment.^20^^,^^25^ The maintenance of this cellular senescence and SASP is promoted by a hyperactive mTOR pathway, the drivers of which are many, with chronic inflammation being a prime example.^26^, ^27^, ^28^, ^29^, ^30^, ^31^, ^32^ Despite these theoretical links, limited data have directly compared the inflammatory transcriptomic profiles of LST, PL, and normal tissue in relation to senescence and their associated risk factors.

Notably, age-related shifts in the colon microbiome toward dysbiosis (termed microb-aging) have been linked to accelerated senescence in model organisms.^33^^,^^34^ In humans, these aging-related microbiome changes, characterized by reduced carbohydrate metabolism and increased prevalence of pathogenic bacteria, including sulfur-reducing bacteria, are associated with SASP and an increased risk of chronic diseases and mortality, manifesting at a younger age in areas with reduced longevity.^35^, ^36^, ^37^, ^38^, ^39^, ^40^, ^41^, ^42^, ^43^, ^44^ Therefore, if present, an increased “microb-aging” signature in LST could potentially induce inflammation, senescence, and an increased mutation rate; however, scarce data exist. In this pilot study, our objectives were to examine the transcriptomic profiles and microbiome signatures of LST compared to paired PL and normal tissue. By utilizing these paired assessments, we aimed to control for interindividual variability. We hypothesize that LST are associated with increased colon inflammation and senescence features compared with paired tissue, which correlates with increased “microb-aging” and a distinct metabolic reprogramming in LST.

## Methods

### The Cohort and Samples

We analyzed formalin-fixed, paraffin-embedded (FFPE) normal and preneoplastic tissues obtained from the UC Davis Pathology Biorepository, an institutional biobank that prospectively collects and catalogs surgical pathology specimens along with associated demographic, clinical, and tumor-specific data. All specimens and accompanying clinical information were deidentified prior to granting access to investigators. The study was conducted under an exempt institutional review board protocol. Cases were selected as a consecutive convenience sample of eligible patients with LST treated between May 2022 and January 2024. Archived tissues were included if they met the following criteria: (1) endoscopic evidence of an LST with histopathologic confirmation; (2) availability of paired normal mucosa or PL tissue for comparison; (3) adequate tissue preservation; and (4) availability of relevant clinical annotation. Cases with carcinoma on the final pathology were excluded. Our resulting cohort included 17 LST, 15 PL, and 4 normal samples from 15 subjects (demographics and tumor characteristics are provided in Table).

**Table:** Demographic and Clinicopathologic Characteristics of the Study Cohort

Sample size: 15 adults, 36 samples (17 LST, 15 PL, 4 normal samples)		
Characteristic	Count	Percentage/range
Females	9	60%
Mean age, in y	69.2	(48–82)
Mean BMI	22.9	(19.6–36.2)
Tissue type		
LST	17	47%
PL	15	42%
Pedunculated polyps subtype	5	14%
Normal	4	11%
Histopathology		
High-grade dysplasia	2	6%
Tubulovillous adenoma	5	14%
Tubular adenoma	17	47%
Sessile serrated adenoma	8	22%
Normal	4	11%
Location		
Cecum	7	22%
Ascending	8	25%
Transverse	5	16%
Descending	7	22%
Rectosigmoid	5	16%

Size	Mean, mm	Range, mm

LST	25.9	15–70
PL	12.9	3–50
Pedunculated polyps subtype	19.1	10–45

Tissue pairing is described in the Methods and Results text.

BMI, body mass index.

### RNA Isolation

Paraffin sections (5 μm thick) were deparaffinized using the Qiagen Deparaffinization Solution before RNA extraction, following the manufacturer’s recommendations with the Qiagen RNeasy FFPE Kit (Qiagen, Hilden, Germany). To remove DNA contamination, we included DNase treatment during RNA extraction. RNA purity and concentration were assessed using the NanoDrop 1000 spectrophotometer (Thermo Fisher Scientific, Waltham, MA). RNA integrity was evaluated using the DV200 metric, determined with the Agilent 2100 BioAnalyzer and the RNA 6000 Nano Total RNA Kit (Agilent Technologies, Santa Clara, CA).

### QuantSeq 3′ mRNA-Seq

RNA libraries were prepared using the QuantSeq 3′ mRNA-Seq Library Prep Kit (Lexogen, Inc., Vienna, Austria) following the manufacturer's standard protocol. This included oligo-dT-primed reverse transcription, second-strand cDNA synthesis, incorporation of unique molecular indices, and library amplification with unique dual indices. Libraries were sequenced on an Illumina NovaSeq X Plus (Illumina, San Diego, CA) using single-end 150 bp reads, generating approximately 20 million reads per sample.

### RNA-Seq Data Analysis

The data were analyzed using the QuantSeq Data Analysis Pipeline Workflow as described in Lexogen’s data analysis manual. Briefly, adapter sequences were trimmed using Cutadapt, and reads were aligned to the GRCh38 reference genome using STAR. Gene annotation was based on GENCODE Human Version 45. After unique molecular index deduplication, gene-level read counts were generated from the BAM file using the HTSeq count.^45^ Transcript counts were then imported into DESeq2^46^ for differential expression analysis and further statistical processing in R. To identify relevant biological pathways, we conducted gene set enrichment analysis (GSEA).^47^ Reconstruction of signaling pathways and functional meanings of differentially expressed transcripts was conducted in QIAGEN Ingenuity Pathway Analysis (IPA; QIAGEN Inc., digitalinsights.qiagen.com/IPA) software. Overall, out of 63,187 transcripts detected in RNA-Seq analysis, 63,060 were mapped by IPA. Considering that multiple transcript identifiers may map to one molecule, the duplicates were resolved by the higher mean intensity of expression between groups. For functional core analysis, we additionally applied cutoffs for expression mean intensity to be above 10 and DESeq2 *P*-value < .05.

### 16S rRNA Amplicon DNA Isolation and Library Preparation

Paraffin sections (5 μm thick) were deparaffinized using the Qiagen Deparaffinization Solution before DNA extraction, following the manufacturer’s recommendations with the QIAamp DNA FFPE Advanced Kit with uracil-N-glycosylase (Qiagen). To remove RNA contamination, we included the RNase treatment during DNA extraction. DNA concentration was assessed using the Qubit dsDNA HS Assay Kit (Invitrogen, Carlsbad, CA).

Samples underwent 16S rRNA gene amplicon high-throughput sequencing using the Element Biosciences AVITI System (Element Biosciences, San Diego, CA). Libraries were prepared following Illumina's 16S Metagenomic Sequencing Library Preparation protocol (Illumina, Part #15044223, Rev. B). Primers targeting the V3–V4 hypervariable region of the 16S rRNA gene, containing Illumina adapter sequences, were utilized.

The first PCR was performed in 50-μL reactions containing 150 ng of purified DNA, 25 μL of 2X KAPA HiFi HotStart ReadyMix (KAPA Biosystems, Wilmington, MA), and 0.5 μM each of forward primer 341F (5′–CCTACGGGNGGCWGCAG–3′) and reverse primer 805R (5′–GACTACHVGGGTATCTAATCC–3′) (Integrated DNA Technologies, Coralville, IA). Thermal cycling was conducted on a Bio-Rad T100 Thermal Cycler (Bio-Rad Laboratories, Hercules, CA) with the following conditions: initial denaturation at 95 °C for 3 minutes; 25 cycles of 95 °C for 30 seconds, 55 °C for 30 seconds, and 72 °C for 30 seconds; and a final extension at 72 °C for 5 minutes.

Subsequent index PCR, library preparation, and sequencing were performed according to standard procedures outlined in Illumina's protocol (Illumina, Part #15044223, Rev. B). The final sequencing library was loaded onto the Element Biosciences AVITI System for 2 × 300 bp paired-end sequencing.

### 16S rRNA Gene Amplicon Analysis

Microbial 16S rRNA gene amplicon sequenced reads data were processed and analyzed using QIIME 2, version 2024.10.^48^ Following the import, the raw demultiplexed reads were trimmed using the *qiime cutadapt trim-paired* plugin.^49^ Next, paired reads were subjected to quality-filtering, joining, and chimera elimination within the denoising process with the DADA2 plugin (*qiime dada2 denoise-paired*).^50^ For the resulting dataset containing amplicon sequence variants (ASVs), a phylogenetic tree was built using FastTree2 and MAFFT alignment within the *qiime phylogeny align-to-tree-mafft-fasttree* pipeline. Taxonomic assignment was conducted with *qiime feature-classifier classify-sklearn* using pre-trained Naïve Bayes classifiers. First, we used SILVA database (release 138) to detect and filter out chloroplasts and mitochondria via *qiime taxa filter-table*. Then, we assigned Greengenes2 taxonomy on the final dataset. Blank and negative control samples were subjected to the same bioinformatic processing. Finally, to determine the differences between experimental groups, ASV tables were collapsed by the genus level (level 6) with *qiime taxa collapse* and subjected to the differential abundance analysis via the analysis of compositions of microbiomes with bias correction (ANCOM-BC) method with *qiime composition ancombc*.^51^ Differential genera were deemed significant with an absolute value of log_2_ fold change > 0.6 and *q*-value < 0.05.

Functional changes of observed ASVs were predicted using the Phylogenetic Investigation of Communities by Reconstruction of Unobserved States 2 (PICRUSt2) pipeline, version 2.4.1. Briefly, PICRUSt2 aligns ASVs to the reference 16S rRNA gene sequences (HMMER, www.hmmer.org) and places them into the phylogenetic reference tree (evolutionary placement algorithm-next generation and genesis applications for phylogenetic placement analysis),^52^^,^^53^ followed by the hidden-state prediction of gene families (castor R package^54^) and, finally, generation of Enzyme Commission metagenomic predictions and tabulation of Enzyme Commission pathways’ inferences and abundances (Minimal set of Pathways^55^ and MetaCyc).^56^

### Statistical Analysis

A differential transcript expression analysis was performed using DESeq2, with lesion type specified as the primary variable in the design formula. All transcriptomic comparisons were performed using paired intrapatient analyses to minimize interindividual variability. All tissue samples were processed and sequenced concurrently using the same extraction, library preparation, and sequencing workflow to minimize technical variability. Because no technical stratification or separate sequencing runs were present, explicit batch correction was not applied. For microbiome analyses, differential abundance testing at the genus level was conducted using ANCOM-BC with lesion type as the primary variable. Functional predictions derived from PICRUSt2 were compared across lesion categories. GSEA, our primary pathway-enrichment analysis, was interpreted using Benjamini-Hochberg false discovery rate (FDR)-adjusted *q*-values, and differential microbiome abundance testing (ANCOM-BC) and the paired immune-cell deconvolution analysis likewise applied Benjamini-Hochberg FDR correction. IPA was used as a complementary, hypothesis-generating tool to aid biological interpretation of the differentially expressed transcripts and was therefore interpreted descriptively without additional multiple-testing correction.

## Results

### GSEA Analysis of Transcript Expression Tables

#### LST exhibit a proliferative, inflammatory, and senescence-associated transcriptomic signature compared to PL

The RNA-Seq transcript data were subjected to GSEA. Compared to all other paired PL, LST showed increased expression of pathways involved in proliferation (MYC transcription factor and E2F targets), the proinflammatory unfolded protein (stress) response, and mTOR activation, as depicted in Figure 1A. In contrast, apical junction genes, a measure of mucosal barrier integrity, and tumor T-cell-mediated killing/immune surveillance (eg, interferon, allograft rejection) were more enriched in PL (Supplementary Table 1). In Chemical and Genetic Perturbations gene sets, colorectal carcinogenesis pathways (eg, Grade Colon And Rectal Cancer: Normalized enrichment score (NES) = 6.65, FDR *q* = 0), stemness pathways (eg, Wong Embryonic Stem Cell Core: NES = 6.12, FDR *q* = 0), senescence (eg, Chicas_RB1_Targets_Senescent: NES = 4.04, FDR *q* = 0), and inflammatory gene sets (eg, Phong TNF Targets Up: NES = 2.26, FDR *q* = 0.005) were enriched in LST, while β-catenin (CTNNB1) signaling was downregulated in PL (eg, Sabates Colorectal Adenoma: NES = −8.39, FDR *q* = 0).

**Figure 1:**
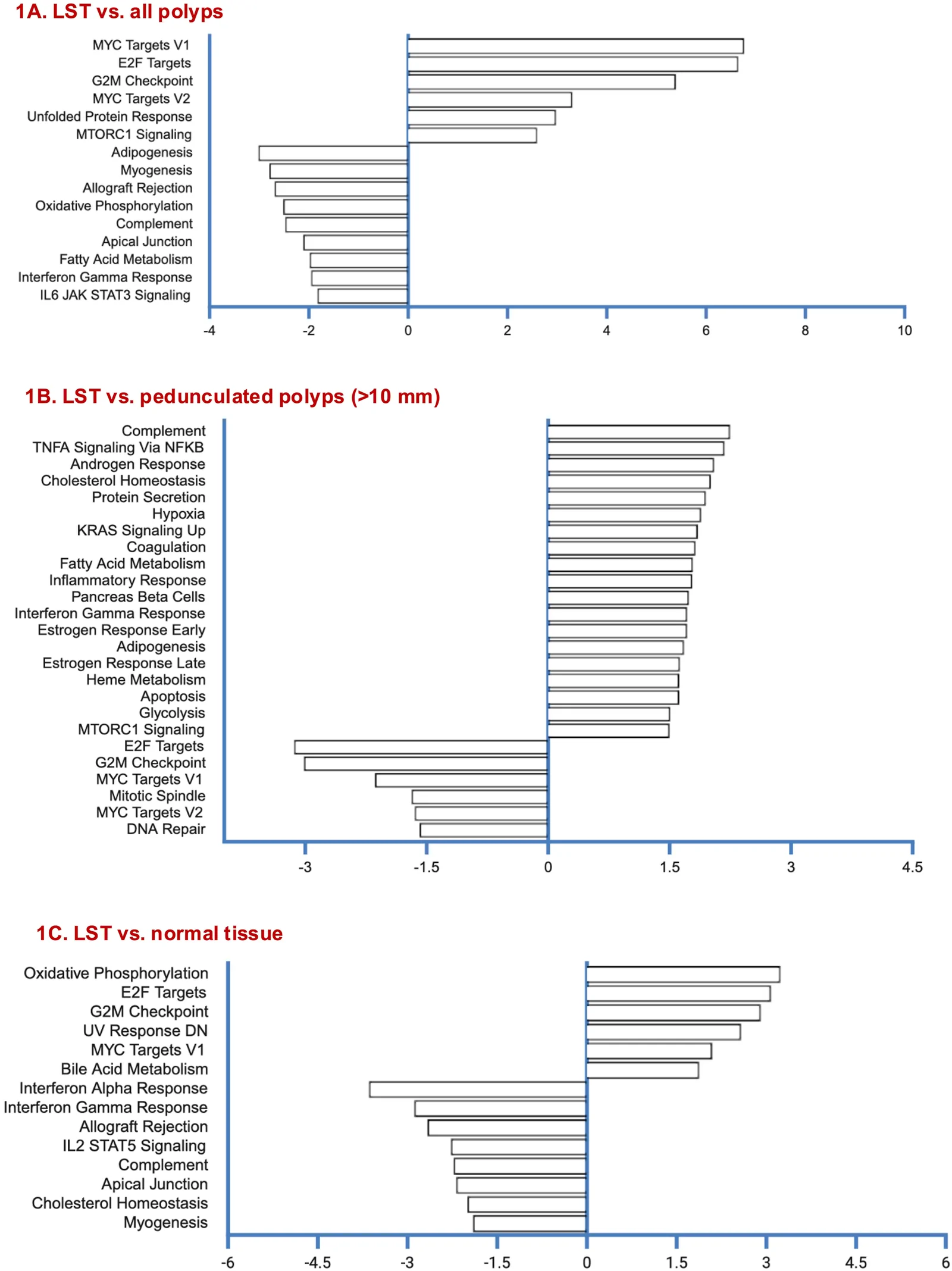
Gene set enrichment analysis of paired RNA-seq samples. (A) LST vs paired protruding lesions (PL). (B) LST vs paired pedunculated polyps (≥10 mm). (C) LST vs paired adjacent normal colonic tissue. The X-axis shows the normalized enrichment score, with positive values indicating enrichment in LST and negative values indicating enrichment in the comparator tissue. Only pathways with FDR-adjusted *q* < 0.05 are shown.

#### Distinct immune activation and DNA damage signaling differentiate LST from size-matched pedunculated polyps

To identify whether these findings are due to tumor size vs nature of LST, we compared LST to the large pedunculated polyp subtype (≥10 mm, Figure 1B). LST showed increased enrichment of gene sets involved in immune activation (eg, TNFA signaling via NF-κB), proliferation, KRAS, metabolic dysregulation, and MTORC1 signaling activation. Pedunculated polyps had higher expression of gene sets involved in cell cycle progression, DNA repair, and proliferation. As described in the Supplementary Table 2, compared to pedunculated polyps, LST also had higher DNA damage signaling (eg, KYNG DNA damage, NES = 1.5, *P*-value < .03). β-catenin (CTNNB1) gene sets were also less enriched in LST than in pedunculated polyps.

#### LST display coordinated activation of metabolic, proliferative, and aging-related gene programs relative to normal tissue

Compared to paired normal tissue (Figure 1C), LST had increased enrichment of gene sets involved in fatty acid metabolism (eg, oxidative phosphorylation, bile acids metabolism), proliferation (eg, E2F, MYC), and DNA damage response (UV Response). Cancer and senescence gene sets were also more enriched in LST as shown in Supplementary Table 3 (eg, SMID Breast Cancer Normal Like: NES = 4.79, FDR *q* = 0, and RODWELL Aging Kidney Up: NES = 4.36, FDR *q* = 0). In contrast, normal paired samples had enrichment of apical junction genes (reduced intestinal permeability) and tumor T-cell-mediated killing/immune surveillance genes (eg, interferon, allograft rejection, IL2).

### Deeper Analysis of the Expression Patterns of LST Polyps vs PL

#### Pathway analysis reveals dominant inflammatory, stress-response, and oncogenic signaling in LST compared to PL

The QIAGEN IPA software was used to further interpret transcript expression patterns in LST relative to PL. The analysis revealed activation of pathways involved in cellular stress, cytokine signaling, and innate and adaptive immune responses in LST (Figure 2A, X-axis). In particular, LST upregulated NOD1/2, S100 family, glycation, macrophage classical activation, CXCR4, IL-17, IL-8, Th2, and neuroinflammation signaling pathways. The pathogen-induced cytokine storm pathway demonstrated the strongest predicted activation (z-score = 3.464; p-overlap = 1.97 × 10^−11^). Activation of tumor microenvironment and tissue factor in cancer pathways further supported enhanced oncogenic signaling in LST. Additional enrichment of hepatic cholestasis signaling pathways reflected shared inflammatory and metabolic transcriptional programs rather than a liver-specific pathology. Overall, these pathways indicate increased immune activation, cellular stress, and tumor-promoting signaling in LST (Figure 2A, Y-axis).

**Figure 2:**
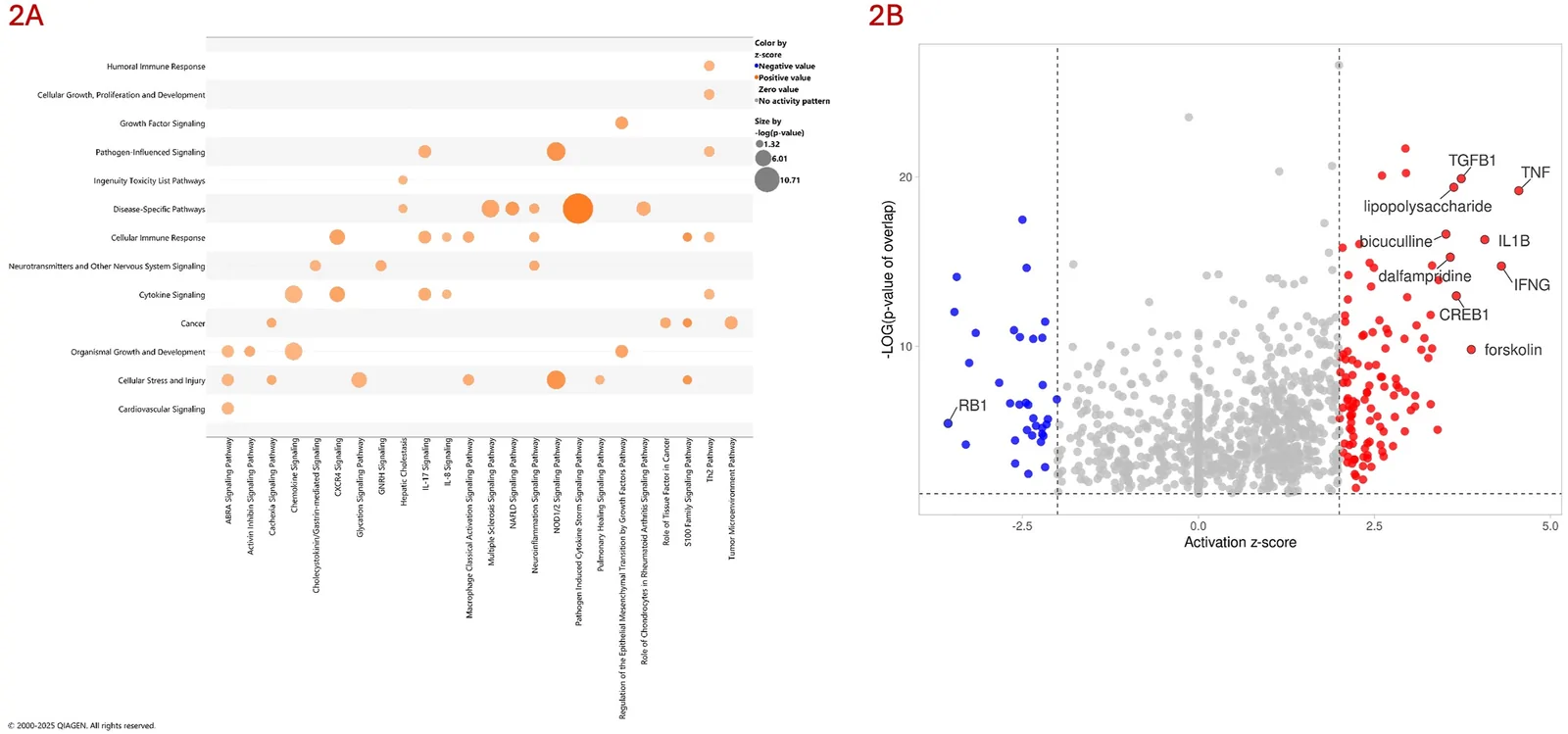
(A) Pathways enriched in LST compared to PL according to transcript expression patterns analyzed by IPA core analysis. Pathway categories are on the Y-axis, while pathway names are on the X-axis. Color indicates activation (shades of orange) of pathways by positive *z*-score (≥2), and the size of each bubble signifies the *P*-value of overlap. No inhibited pathways (*z*-score ≤ −2) were detected. (B) Upstream regulators and their activation status in LST compared to PL according to transcript expression patterns analyzed by IPA core analysis. Activation *z*-scores are on the X-axis (red indicates predicted activation [*z*-score ≥ 2], and blue indicates predicted inhibition [*z*-score ≤ −2]). Negative log of *P*-values of overlap are on the Y-axis. The complete list of activated and inhibited upstream regulators is provided in Supplementary Table 4.

#### Upstream regulator analysis implicates cytokine-driven inflammation and impaired tumor suppression in LST compared to PL

An upstream regulator analysis identified the principal molecules predicted to drive the transcriptomic differences between LST and PL (Figure 2B, Supplementary Table 4). The highest activation *z*-scores (≥4) were observed for cytokines TNF, IFNG, and IL1B. Additional activated cytokine regulators included OSM, LIF, EPO, TNFSF13B, IL15, IL17A, IL3, C5, PRL, IL13, CSF2, and CSF1 (*z*-scores ≥ 2). Lipopolysaccharide (LPS) was also among the regulators with the highest *z*-scores, supporting a microbiota-associated inflammatory response in LST. In contrast, the tumor suppressor RB1 exhibited the strongest predicted inhibition, suggesting a loss of growth suppressive signaling. Multiple oncogenic regulators, including EGF, ERK1/2, MAPK8, RELA, NF-κB, TGF-β/TGFB1, VEGFA, and MMP9, were also predicted to be activated, consistent with enhanced proliferative, inflammatory, and tissue-remodeling programs in LST. Predicted activation of RELA, ERK1/2, and STAT3 further supports activation of the SASP in LST. Consistent with this observation, IPA pathway mapping demonstrated activation of multiple senescence-associated regulatory networks, including ERK/MEK, FOXO3, SMAD3, TGF-β, and TP53 signaling, together with inhibition of NFAT5 (Supplementary Figure 1).

#### LST demonstrate enhanced immune activation and tissue remodeling compared with pedunculated polyps

In contrast to pedunculated polyps, LST demonstrated activation of canonical pathways associated with inflammation, immune activation, oncogenic signaling, and cellular stress (Figure 3A). Activated pathogen-induced cytokine storm, chemokine signaling, macrophage activation signaling pathways, and dendritic cell maturation support a robust inflammatory immune response. Upregulated IL-8 and IL-17A signaling pathways with CXCR4, TCR signaling, T-cell and B-cell receptor signaling, and the Th2 pathway further suggest an upregulated innate and adaptive immune response. Focal adhesion kinase (FAK) signaling, regulation of the epithelial-mesenchymal transition by growth factors, wound healing, and molecular mechanisms of cancer pathways were also activated, consistent with tissue remodeling, fibrosis, and potential cancer progression. In contrast, CTLA4 signaling in cytotoxic T lymphocytes and RhoGDI signaling were inhibited, suggesting altered immune regulation and intracellular signaling compared with pedunculated lesions.

**Figure 3:**
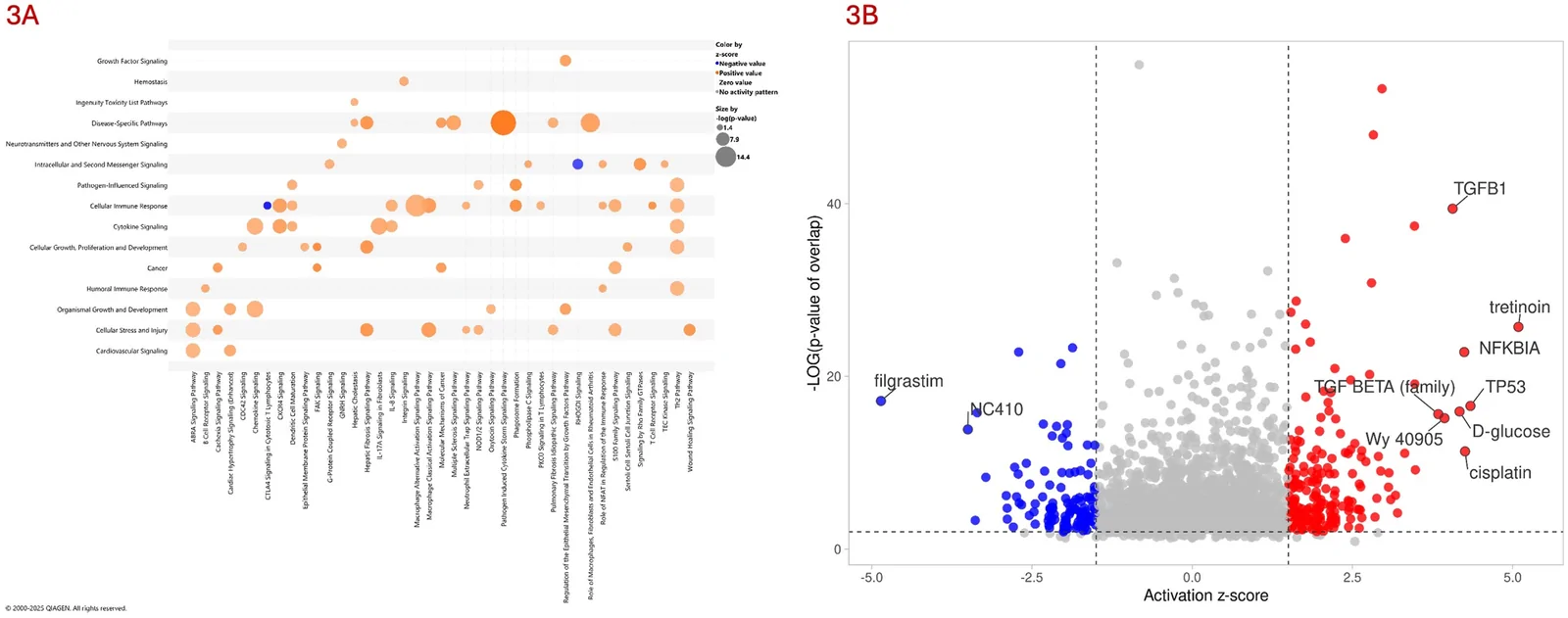
(A) Pathways enriched in LST compared to pedunculated polyps according to transcript expression patterns analyzed by IPA core analysis. Pathway categories are on the Y-axis, while Pathway names are on the X-axis. Color indicates activation (shades of orange) of pathways by positive *z*-score (≥2.5), and the size of each bubble signifies the *P*-value of overlap. No inhibited pathways (*z*-score ≤ −2.5) were detected. (B) Upstream regulators and their activation status in LST compared to pedunculated polyps according to transcript expression patterns analyzed by IPA core analysis. Activation *z*-scores are on the X-axis (red indicates predicted activation [*z*-score ≥ 2] and blue indicates predicted inhibition [*z*-score ≤ −2]). Negative log of *P*-values of overlap is on the Y-axis. The complete list of activated and inhibited upstream regulators is provided in Supplementary Table 5.

#### Cancer-associated and metabolic upstream regulators distinguish LST from pedunculated lesions

The upstream regulator analysis further supported activation of multiple oncogenic signaling networks in LST compared with pedunculated polyps (Figure 3B, Supplementary Table 5). Activated regulators included inflammatory and oncogenic mediators such as IL1B, JAK3, MAPK8, MMP9, NFκBIA, PTK2, TGF-β/TGFBR2, TP53, and VEGF, whereas HDAC3, RB1, KRAS, MYC, and PRKCI were predicted to be inhibited. Activation of D-glucose, FGF19, FOXO3, KEAP1, mTOR, and VEGF further supported metabolic reprogramming consistent with Warburg metabolism and an activated tumor microenvironment. Among the most significant upstream regulators were LPS, TNF, and IFNG, supporting a microbiota-associated inflammatory response in LST.

#### Loss of homeostatic signaling and activation of oncogenic pathways characterize LST relative to normal tissue

Compared with normal tissue, LST demonstrated broad suppression of pathways involved in cellular homeostasis, intracellular signaling, growth and development, and neurotransmitter signaling (Figure 4A). Among the top-activated pathways were cell cycle control of chromosomal replication, cyclins and cell cycle regulation, NOD1/2 signaling, and LPS/IL-1-mediated inhibition of the RXR function, supporting enhanced proliferative and inflammatory signaling and implicating microbial-derived inflammatory stimuli in LST biology. Conversely, cardiac hypertrophy, serotonin receptor, retinoic acid receptor, and G protein-coupled receptor-mediated nutrient-sensing pathways were among the most strongly inhibited pathways relative to normal tissue. Additional inhibition of AMP-activated protein kinase signaling, with activation of the Warburg effect, IL-17 signaling, and macrophage alternative activation pathways, further supports metabolic reprogramming, immune activation, and a tumor-promoting phenotype in LST.

**Figure 4:**
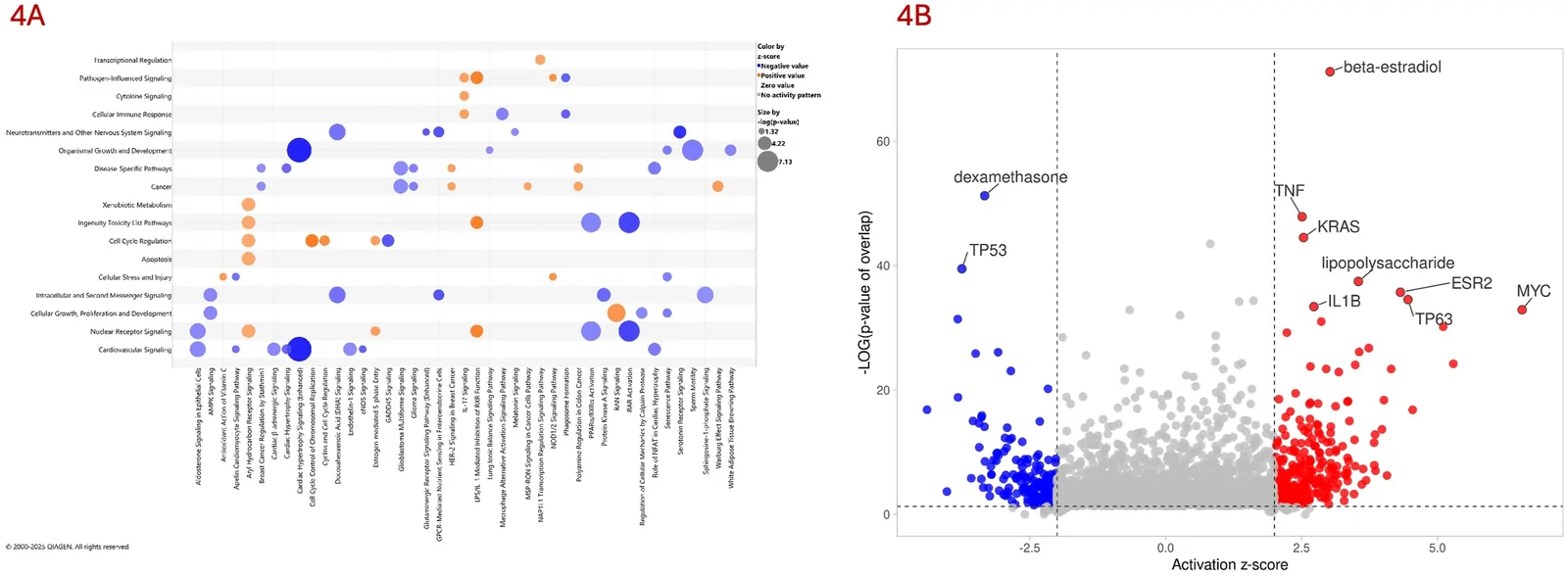
(A) Pathways enriched in LST compared to normal tissue according to transcript expression patterns analyzed by IPA core analysis. Pathway categories are on the Y-axis, while pathway names are on the X-axis. Color indicates activation (shades of orange) of pathways by positive *z*-scores (≥2) or inhibition (shades of blue) with negative *z*-scores (≤−2). The size of each bubble signifies the *P*-value of overlap with observed differential transcripts. (B) Upstream regulators and their activation status in LST compared to normal tissue samples according to transcript expression patterns analyzed by IPA core analysis. Activation *z*-scores are on the X-axis (red indicates predicted activation (*z*-score ≥ 2), and blue indicates predicted inhibition (*z*-score ≤ −2). Negative log of *P*-values of overlap is on the Y-axis. The complete list of activated and inhibited upstream regulators is provided in Supplementary Table 6.

#### Transcriptomic profiles of LST reflect cytokine activation and downregulation of key tumor suppressor pathways when compared to normal tissue

The upstream regulator analysis further identified a broad inflammatory and oncogenic regulatory program distinguishing LST from normal tissue (Figure 4B, Supplementary Table 6). Activated upstream cytokines included CSF2, IL22, EDN1, IL17A, CD40LG, IL1B, WNT3, TNF/TNFSF10/TNFSF12, IL3, IL1A, C5, CCL2, LIF, and CXCL12. MYC and LPS were also among the highest-ranked activated upstream regulators, supporting coordinated proliferative and microbiota-associated inflammatory signaling. In contrast, TP53, IL10RA, CDKN2A, RB1, and APC were among the most strongly inhibited upstream regulators, consistent with reduced tumor suppressor activity. Activation of WNT3A, CTNNB1, FZD7, and the BCL9/CREBBP/EP300/CTNNB1/LEF/TCF transcriptional complex further supported enhanced Wnt/β-catenin signaling in LST compared to normal tissue. Activation of AKT, CCND1, CHEK1, E2F, ERK, KRAS, JUN, NF-κB (NFKB1/RELA), STAT3, and reactive oxygen species, together with inhibition of CDKN2A, IFN type I, NF1, RB1, and TP53, supports disruption of canonical senescence regulatory pathways while simultaneously promoting activation of SASP (Supplementary Figure 2). Collectively, these findings are consistent with a chronically inflamed, tumor-promoting microenvironment that favors LST progression.

#### Digital cytometry supports adaptive immune enrichment in LST

To further explore the immune populations underlying the inflammatory transcriptomic profile, we performed exploratory digital cytometry using microenvironment cell populations-counter in patients with paired LST and PL. Compared with paired PL, LST demonstrated increased B-cell, T-cell, and CD8+ T-cell signatures, with concordant trends toward increased cancer-associated fibroblast and myeloid dendritic-cell signatures. These findings provide orthogonal support for the adaptive immune and inflammatory programs identified by GSEA and IPA and are consistent with a chronically inflamed, rather than an immune-depleted, microenvironment. Because immune-cell composition was computationally inferred from bulk FFPE-derived RNA-seq data, these results should be interpreted as exploratory estimates of relative immune-cell signature enrichment rather than direct measurements of immune-cell abundance (Supplementary Table 7 and Supplementary Figure 3).

### Analysis of Microbial Ecosystem Associated With LST

#### Predicted microbial functions indicate reduced carbohydrate fermentation and altered sulfur metabolism in LST

To investigate whether transcriptomic differences were accompanied by alterations in microbial function, we performed 16S rRNA gene sequencing, followed by a PICRUSt2 functional prediction. No significant functional differences were identified between LST and all non-LST lesions. In contrast, a comparison with pedunculated polyps identified enrichment of 2,3-cyclic-nucleotide 3-phosphodiesterase and allantoate deiminase in LST (Figure 5A). More pathway differences in LST were observed compared to normal tissues (Figure 5B). LST-associated microbiota demonstrated reduced pathways involved in carbohydrate fermentation and glycolytic metabolism, including hexitol fermentation and glycol degradation, together with reduced geranylgeranyldiphosphate biosynthesis via the mevalonate pathway. Predicted enzyme analyses further suggested reduced carbohydrate utilization, including decreased pyruvate oxidase, ribulokinase, glycolaldehyde dehydrogenase, and lactaldehyde dehydrogenase activity (Figure 5C). Predicted sulfur metabolism was also altered, with reduced assimilatory sulfite reductase (ferredoxin), homocysteine S-methyltransferase, cystathionine metabolism, and sulfite dehydrogenase, suggesting impaired microbial sulfur assimilation and transsulfuration. Conversely, sulfopropanediol 3-dehydrogenase was enriched, indicating altered sulfur compound metabolism within the LST-associated microbiome.

**Figure 5:**
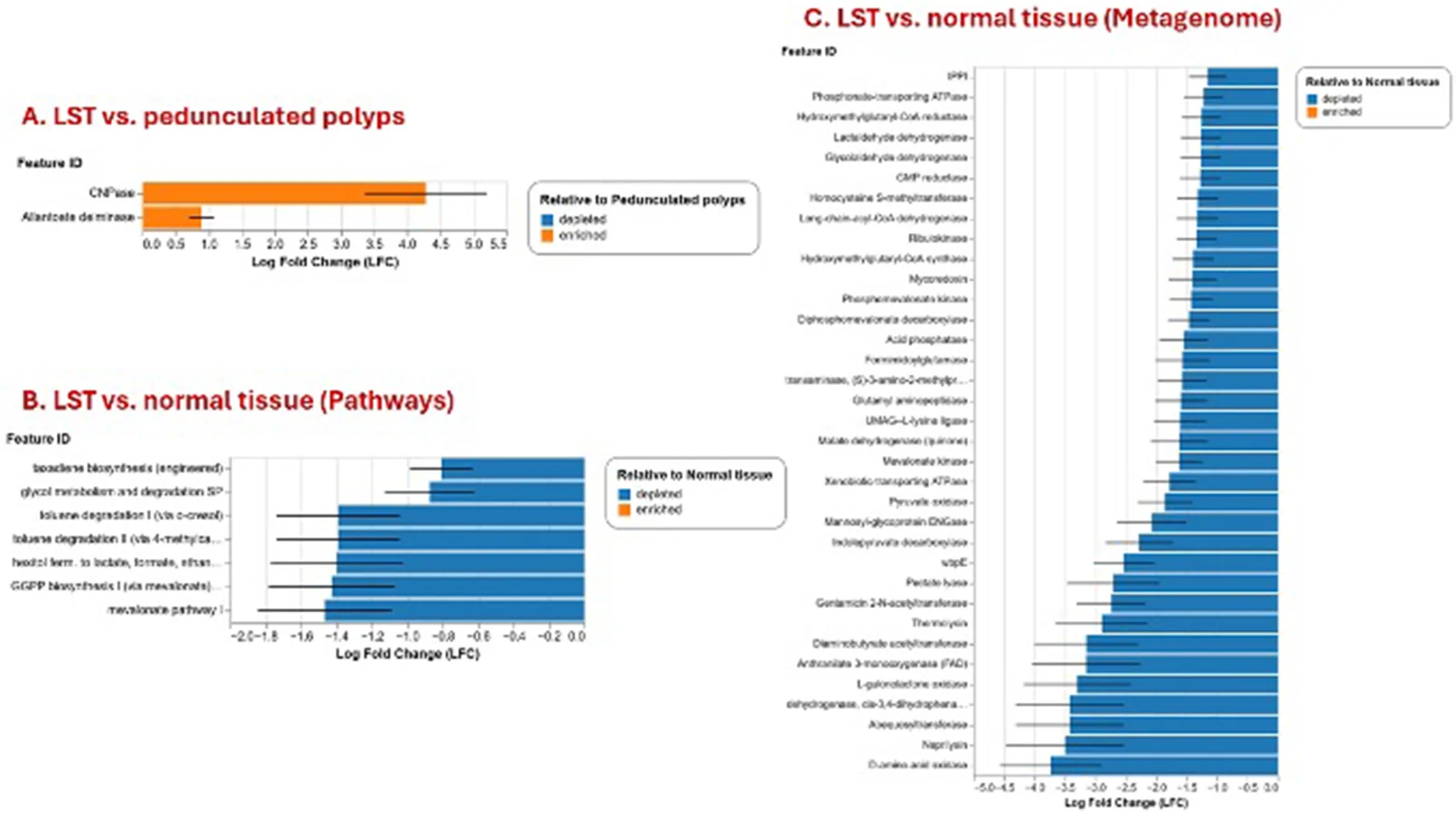
Bacterial functions associated with LST microbiota predicted by PICRUSt2. A differential analysis of Enzyme Commission pathways was conducted using ANCOM-BC in LST compared to paired pedunculated polyps (A) or normal tissue (B). Enzyme Commission metagenome of LST vs paired normal tissue is displayed in panel C. Only features reaching *q* < 0.05 are depicted.

#### Taxonomic differences and correlation with transcriptomic data

The predicted functional differences were accompanied by enrichment of *Fusobacterium* and depletion of *Moraxellaceae* in LST compared to PL (Figure 6). Relative to normal tissue samples, LST had lower levels of *Lactococcus*, *Ga0074137*, and *Alkanindiges*. These taxonomic shifts were consistent with the functional alterations predicted by PICRUSt2, including reduced carbohydrate fermentation and altered sulfur metabolism. Finally, we identified a moderate correlation between the expression of TNF genes in LST tissue and LST *Fusobacterium* DNA levels (Figure 6D, Supplementary Table 8), supporting an association between *Fusobacterium* enrichment and inflammatory signaling in LST.

**Figure 6:**
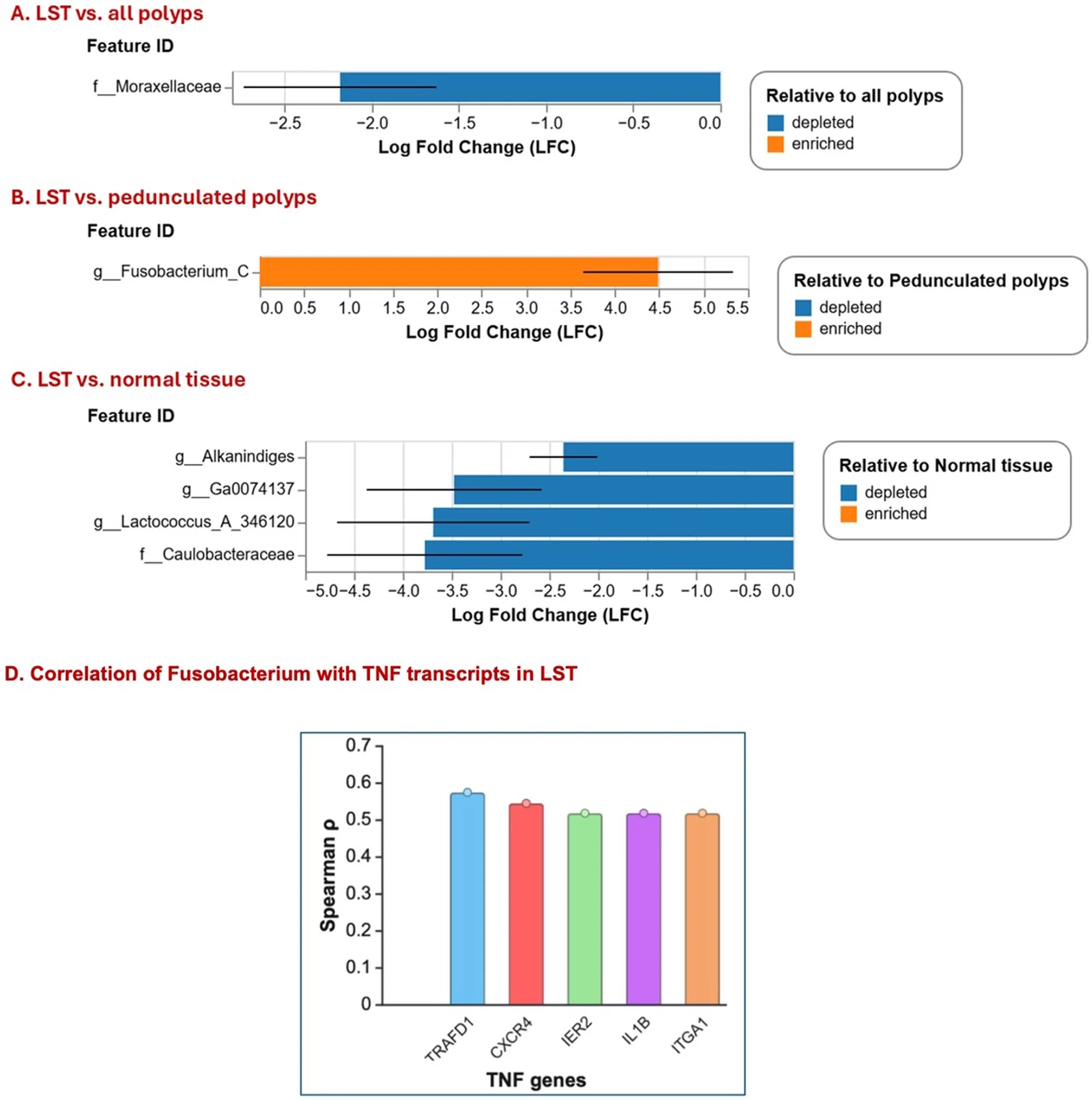
Bar plots of ANCOM-BC results of differential flat polyp microbiota on a genus level compared to all lesions (A), pedunculated (B), or normal tissue (C). Panel D depicts the correlation between the abundance of *Fusobacterium* and the expression of TNF genes in LST samples (*P* < .05).

## Discussion

This study provides a comprehensive characterization of the host transcriptomic and microbial landscape of LST, demonstrating that these lesions possess a distinct inflammatory, metabolic, and senescence-associated phenotype compared with paired PL and normal tissue. LST were characterized by enrichment of *Fusobacterium*, reduced microbial carbohydrate fermentation capacity, and activation of TNF/NF-κB, mTOR, MYC/E2F, DNA damage, and senescence-associated pathways, together with exploratory evidence of increased adaptive immune-cell signatures. The strong enrichment of stemness-associated gene signatures further supports a shift toward an undifferentiated, high-grade neoplastic state. This is consistent with prior investigations in an unpaired format, which identified reduced butyrogenic bacteria and increased biofilm-forming/sulfur-reducing bacteria that promote senescence in fecal samples from adults with vs without LST.^57^ Collectively, these findings support a model in which microbial dysbiosis, chronic inflammation, and senescence-associated signaling cooperate to establish a tumor-promoting microenvironment that may contribute to the accelerated malignant potential of LST.

A defining characteristic of LST is their nonprotruding, laterally spreading growth pattern, which likely reflects distinct molecular and structural adaptations compared with conventional PL. Prior transcriptional studies have identified increased expression of Wnt/β-catenin/c-MYC and axonal guidance genes in LST compared with PL.^14^^,^^58^, ^59^, ^60^, ^61^ Membrane-localized β-catenin has been proposed to preserve apical-basal polarity and may contribute to the characteristic lateral morphology of these lesions.^62^ Furthermore, the hyperactivation of matrix metalloproteinases (eg, MMP9), TNF (inflammation), and CXCR4 signaling (chemokine/migration) likely facilitates local tissue remodeling and cell migration, whereas the observed reduced enrichment of tight junctions compromises epithelial barrier integrity and promotes paracellular transport of proinflammatory bacterial metabolites.^14^^,^^63^

Indeed, our GSEA results demonstrated significant depletion of the Hallmark Apical Junction gene set (NES = −4.18) in LST compared with pedunculated polyps, indicating disruption of epithelial organization and barrier integrity. Together with activation of MMP9, TNF, and CXCR4 signaling, these findings are consistent with extracellular matrix remodeling, increased epithelial permeability, and a microenvironment permissive to chronic inflammatory signaling. Within this context, GSEA and IPA demonstrated coordinated activation of inflammatory, proliferative, and senescence-associated pathways in LST, including MYC/E2F, mTOR, Wnt/β-catenin, and multiple cytokine signaling programs, together with reduced expression of genes involved in immune surveillance. Exploratory digital cytometry further suggested increased B-cell, T-cell, and CD8+ T-cell signatures in LST relative to paired PL. Considered alongside reduced interferon signaling and cytotoxic immune pathways, these findings suggest that LST are characterized by persistent adaptive immune-cell recruitment occurring within a chronically inflamed but functionally dysregulated microenvironment rather than an immune-desert phenotype.

Importantly, these molecular differences persisted when LST were compared with high-risk pedunculated polyps, indicating that the observed inflammatory phenotype is unlikely to be explained solely by lesion size. IPA demonstrated robust activation of pathogen-induced cytokine storm (z-score = 5.37), oncostatin M signaling (z-score = 5.20), and multiple inflammatory cytokine pathways, including TNF, IFNG, IL1B, IL17, and LPS-associated signaling, together with activation of ERK/MAPK and TGF-β pathways and inhibition of the tumor suppressor RB1. Collectively, these findings support a chronically inflamed, tumor-promoting microenvironment characterized by persistent cytokine signaling, stromal remodeling, and disrupted tumor-suppressive pathways.

Interestingly, exploratory deconvolution also suggested a trend toward increased cancer-associated fibroblast signatures, consistent with an evolving senescence-associated stromal niche. Senescent fibroblasts have been shown to promote colorectal tumorigenesis through SASP signaling and to impair CD8+ T-cell function through metabolic reprogramming, providing a biologically plausible mechanism linking epithelial senescence, stromal remodeling, and ineffective antitumor immunity.^26^^,^^32^ Collectively, these findings support a model in which persistent microbial stimulation, chronic inflammatory signaling, adaptive immune-cell recruitment, and stromal remodeling cooperate to promote the accelerated malignant potential of LST.

Moreover, strong evidence implicates the colonic microbiome in senescence and CRC development through chronic inflammation and metabolic reprogramming. In the present study, LST-associated microbiota demonstrated reduced pathways involved in carbohydrate fermentation together with altered sulfur metabolism, indicating a shift toward microb-aging. Consistent with these functional changes, LST exhibited depletion of beneficial taxa, including *Lactococcus*, suggesting reduced microbial capacity for short-chain fatty acid (SCFA) production and maintenance of epithelial homeostasis. Reduced carbohydrate fermentation and SCFA production may compromise epithelial barrier integrity, thereby facilitating chronic inflammation and tumor-promoting signaling. In contrast, *Fusobacterium* was enriched in LST compared with paired PL. *Fusobacterium* is an adherent, invasive, and proinflammatory bacterium whose abundance is influenced by dietary fiber intake and has been repeatedly associated with colorectal carcinogenesis.^64^^,^^65^ In addition to activating inflammatory pathways through LPS and other virulence factors, *Fusobacterium* contributes to sulfur metabolism and biofilm formation, providing a biologically plausible mechanism linking microbial dysbiosis with persistent mucosal inflammation.^66^^,^^67^ The positive correlation between *Fusobacterium* abundance and tissue TNF expression further supports an association between microbial dysbiosis and inflammatory signaling in LST. Together, these findings support a model in which age- and diet-associated microb-aging promotes a microbial ecosystem characterized by reduced SCFA production and enrichment of proinflammatory bacteria. Such changes may increase epithelial permeability, activate NF-κB- and mTOR-associated inflammatory signaling, and promote DNA damage, thereby creating conditions that favor neoplastic progression.^29^^,^^30^^,^^68^, ^69^, ^70^, ^71^, ^72^, ^73^, ^74^, ^75^, ^76^, ^77^, ^78^, ^79^, ^80^, ^81^, ^82^, ^83^, ^84^, ^85^, ^86^, ^87^, ^88^, ^89^ Although these observations do not establish causality, they suggest that host-microbiome interactions may contribute to the unique inflammatory and senescence-associated phenotype observed in LST.

Our study has several strengths, including the use of paired lesion and the integration of transcriptomic and microbiome data to identify host-microbe interactions. Complementary pathway analyses further enabled comprehensive characterization of the LST microenvironment. However, several limitations remain. First, the relatively small sample size, inherent to pilot multi-omic studies of this uncommon lesion, limited subgroup analyses by sex, lesion size, dysplasia grade, and clinical outcomes and precluded incorporation of additional clinical covariates into the primary statistical models. Second, orthogonal validation of individual transcripts by qPCR or Western blot was not feasible because of the limited residual FFPE tissue remaining after RNA extraction. Third, IPA and immune-cell deconvolution were performed as complementary exploratory analyses to facilitate biological interpretation rather than as primary inferential analyses, and bulk RNA-seq cannot definitively assign inflammatory transcripts to specific cell populations; therefore, spatial transcriptomic and single-cell approaches will be needed to resolve the cellular architecture of the LST microenvironment. Finally, the absence of longitudinal follow-up data precluded the evaluation of temporal relationships between microbial dysbiosis, host transcriptomic alterations, and lesion progression, while the use of archived FFPE tissue may introduce RNA degradation and technical variability. Together, these limitations underscore the need for prospective validation in larger fresh-frozen cohorts.

## Conclusion

In summary, our findings demonstrate that LST possess a distinct host-microbiome signature characterized by chronic inflammatory signaling, metabolic reprogramming, senescence-associated pathways, and microbial dysbiosis. Together, these features support a model in which persistent epithelial-microbial interactions contribute to a chronically inflamed, tumor-promoting microenvironment that may underlie the accelerated malignant potential of LST. These findings provide a biological framework for future studies evaluating host-directed and microbiome-targeted prevention strategies, including modulation of inflammatory signaling and restoration of a health-associated microbial ecosystem. Prospective validation in larger cohorts integrating spatial transcriptomics, metagenomics, and metabolomics will be essential to define the mechanistic basis of these interactions and identify biomarkers and therapeutic targets for LST prevention.

## Supplementary Materials

Supplementary Figures

Supplementary Table s

Extended PDF
