# Supplementary material for: A Distinct Host-Microbiome Signature Underlies the Accelerated Malignant Potential of Colorectal Laterally Spreading Tumors
Source: Gastro Hep Adv. 2026 Aug 21;5(12):101102. doi: 10.1016/j.gastha.2026.101102 (PMC13625876; doi:10.1016/j.gastha.2026.101102)
Supplement: Supplementary file 1 [file mmc1.pdf]

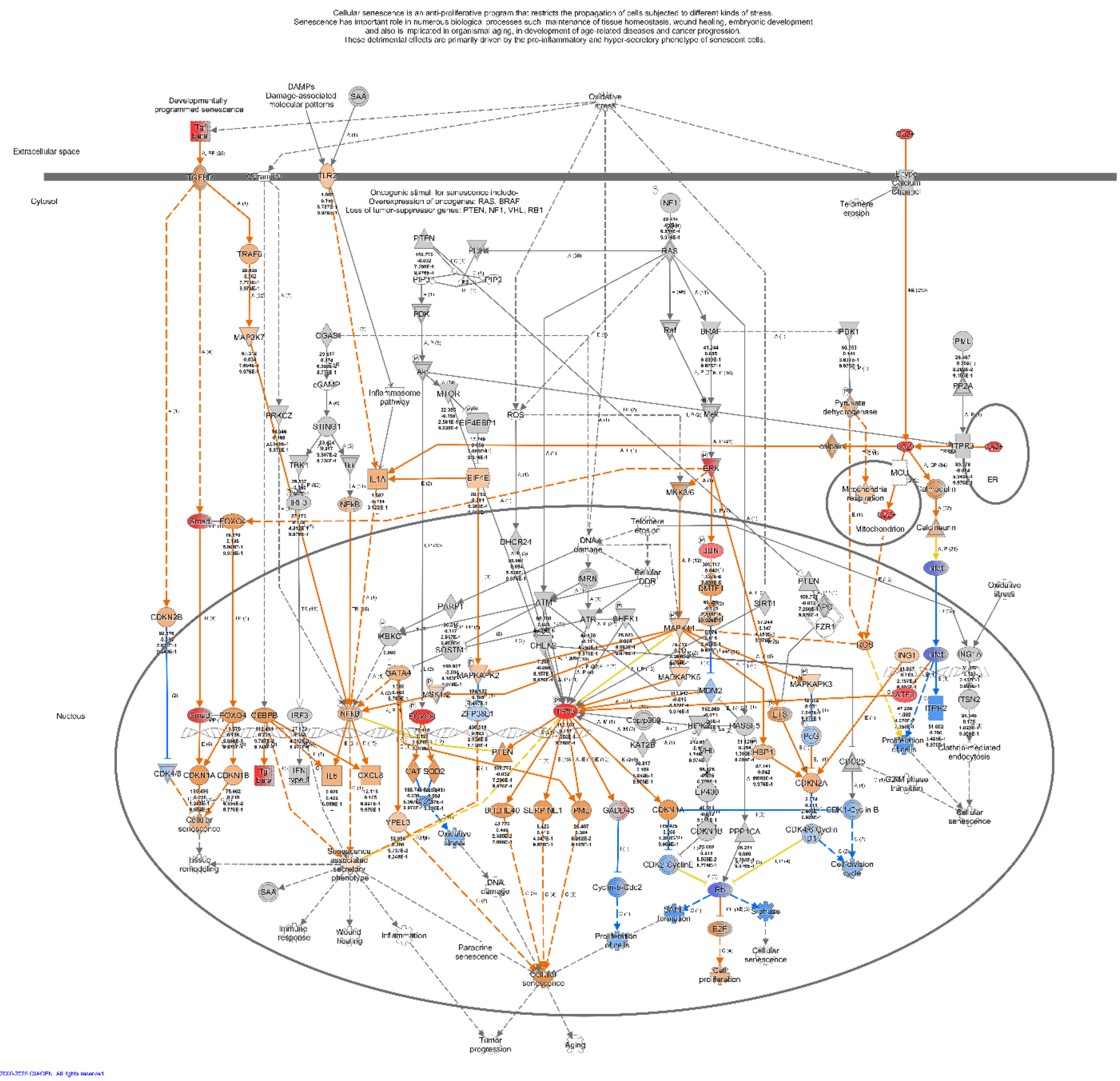

Figure S1. Senescence pathway overlapped with observed DETs and their significant upstream regulators in LST compared to other polyps according to transcript expression patterns analyzed by IPA Core Analysis.

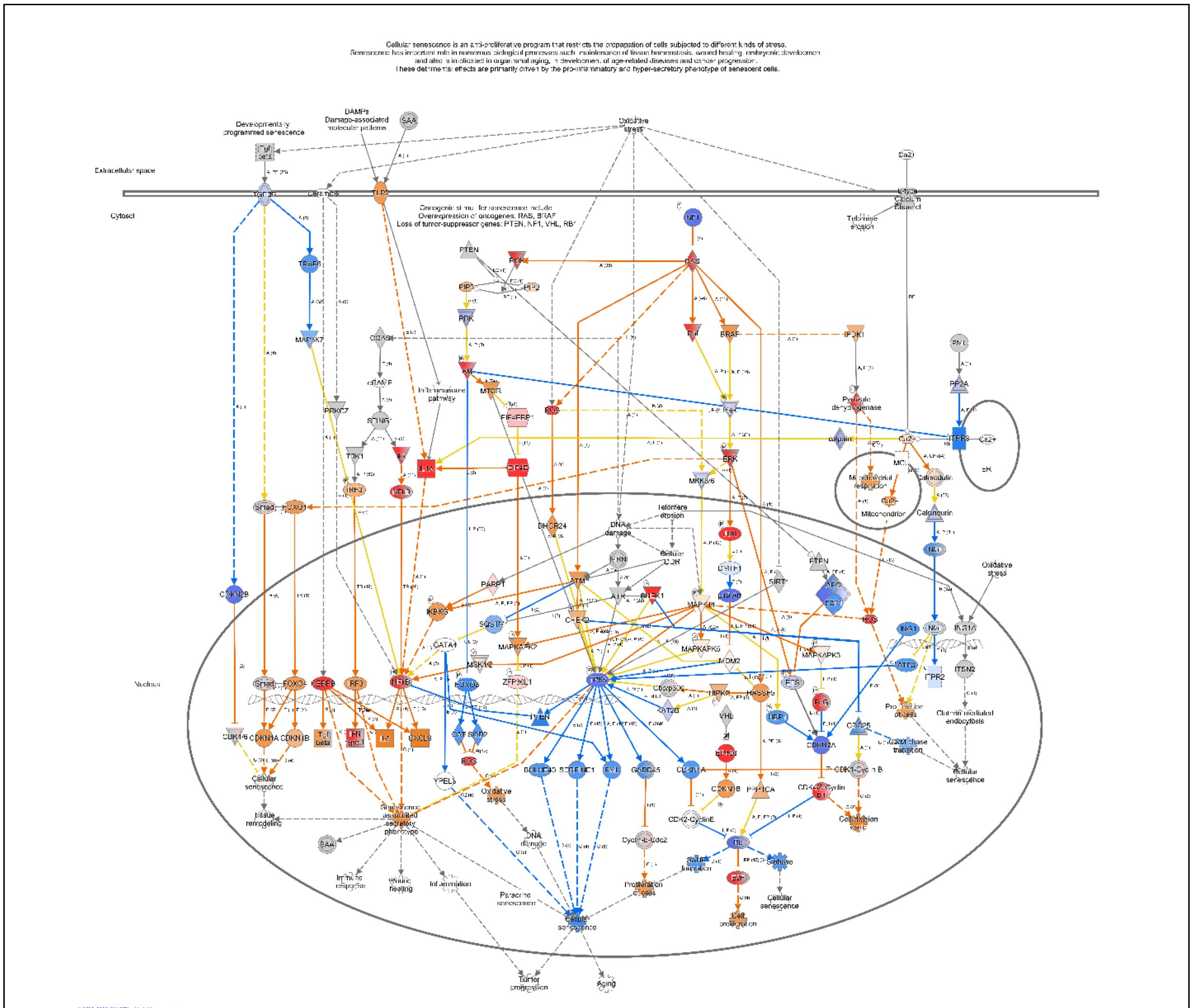

Cellular senescence is an anti-proliferative program that restricts the propagation of cells subjected to different kinds of stress.
These detrimental effects are primarily driven by the pro-inflammatory and hyper-secretory phenotype of senescent cells.
DAMPs
Damage-associated
molecular patterns
SAA
Oxidative
stress
Developmentally
programmed senescence
Extracellular space
Cytosol
Nucleus
Oncogenic stimuli for senescence include:
Overexpression of oncogenes: RAS, BRAF
Loss of tumor-suppressor genes: PTEN, NF1, VHL, RB1
Mitochondrion
ER
Cellular
senescence
Tumor
progression
Aging

A

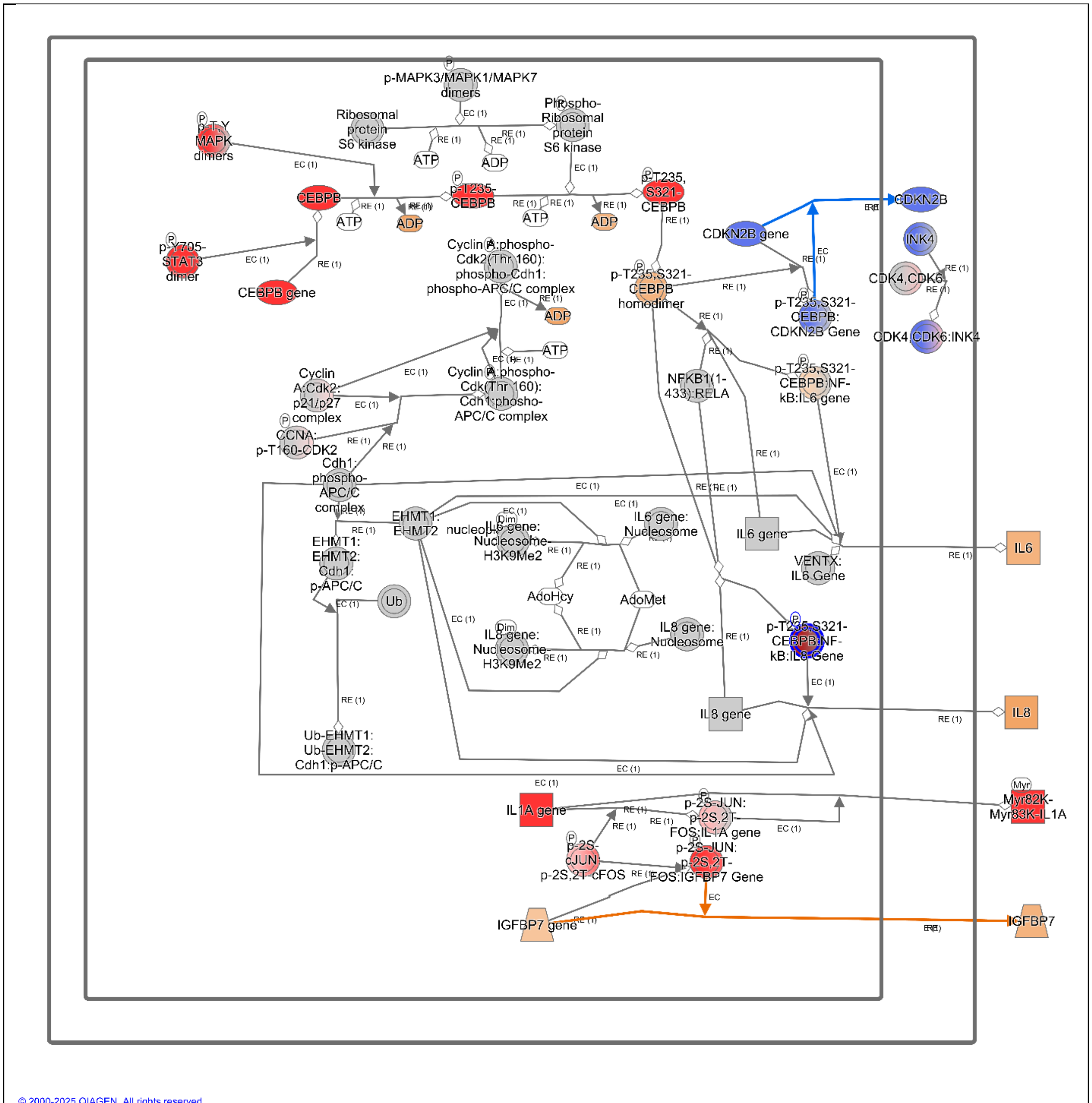

B

Figure S2. Senescence pathway (**A**) and senescence-associated secretory phenotype (SASP) pathway (**B**) overlapped with observed DETs and their significant upstream regulators in LST compared to normal tissue samples according to transcript expression patterns analyzed by IPA Core Analysis.

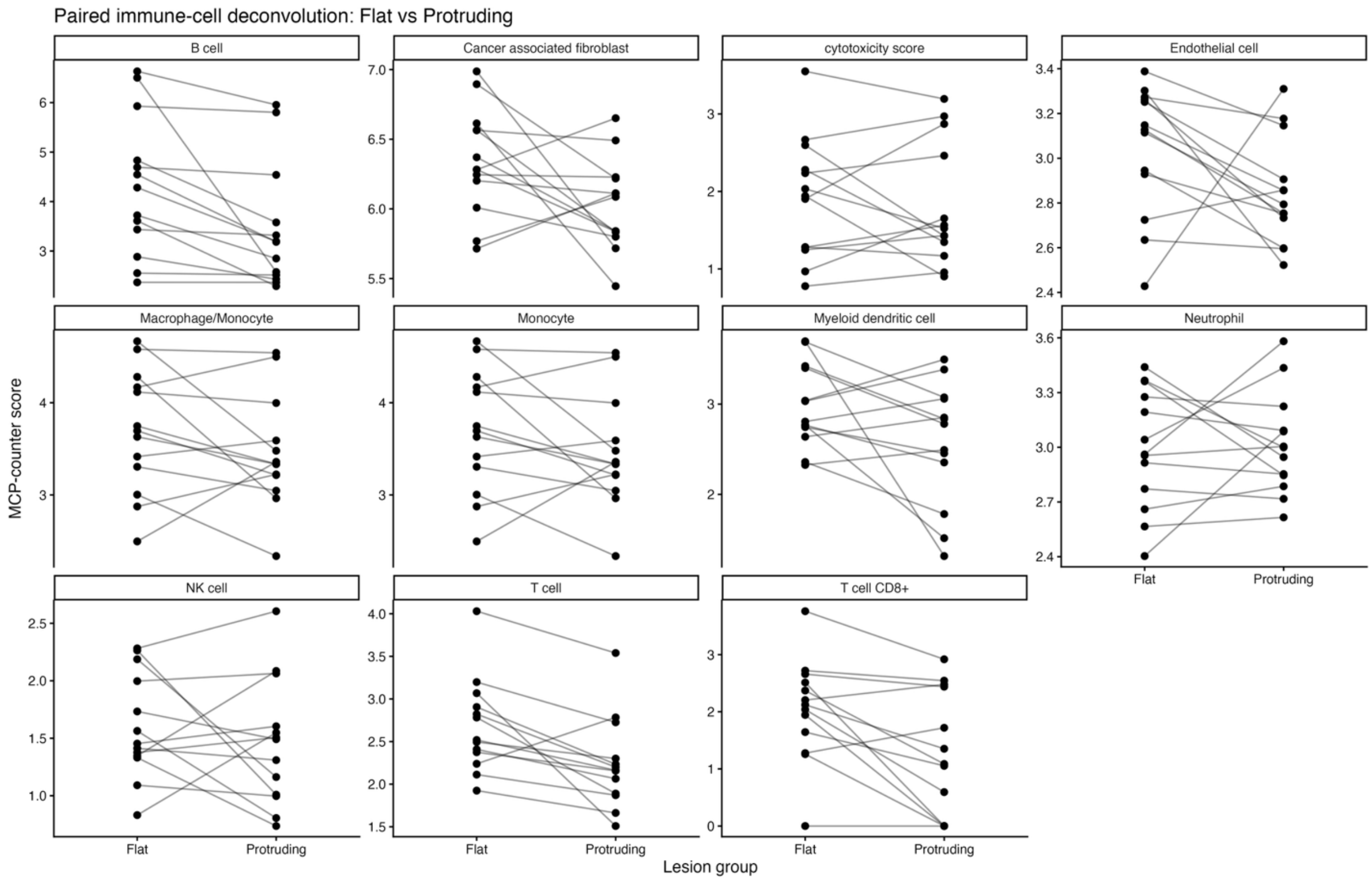

Figure S3. Exploratory immune-cell deconvolution of bulk RNA-seq data using MCP-counter comparing paired flat (LST) and protruding lesions (one lesion of each type per patient). Lines connect paired samples from the same patient. MCP-counter scores represent relative enrichment of immune and stromal cell populations inferred from transcriptomic data and are intended as complementary, hypothesis-generating analyses rather than direct measurements of immune-cell abundance.
